# Effect of team-building sports games on the resilience of Chinese rural children: evidence from Nanxian county, Hunan province

**DOI:** 10.3389/fped.2025.1552597

**Published:** 2025-03-05

**Authors:** Bo Liu, Jizhi You, Yunxiang Fan, Yunping Xia, Hui Wang, Xiang Zhang, Yang Zhang

**Affiliations:** ^1^ College of Physical Education, Hunan Normal University, Changsha, China; ^2^ College of Optical and Electronic Technology, China Jiliang University, Hangzhou, China; ^3^ College of Educational Science, Hunan Normal University, Changsha, China; ^4^ Independent Researcher, Windermere, FL, United States

**Keywords:** exercise, mental health, group sports, physical activity, physical contact, touch

## Abstract

**Purpose:**

Participation in competitive sports has been shown to enhance children's mental health. However, evidence regarding the impact of group sports within traditional physical education on children's mental development is scarce. This study designed a team-building-focused physical education program and examined its effects on the resilience of rural students.

**Methods:**

A total of 86 rural elementary school students (mean age: 11 years) were cluster-randomized into the intervention and control groups. Both groups participated in regular physical education classes three times per week. In the intervention group, the first 15-min warm-up period was replaced with sports games. From a social perspective, all games subtly encourage children to forge new social relationships by engaging in physical contact, interacting with new team members, and collaborating to achieve shared goals in a sportsmanlike manner. Resilience was assessed using the Resilience Scale for Chinese Adolescents before and after the 12-week intervention.

**Results:**

After 12 weeks, the intervention group showed significant improvements in affect control, positive thinking, and help-seeking compared to the control group. Overall, both the individual and supportive dimensions of resilience improved following participation in team-building sports games.

**Conclusions:**

Whether winning or losing as a team member, these experiences positively enhance children's emotional regulation, their understanding of their social roles within a team, and the development of help-seeking and concern for a distressed other. As a result, children may build new and broader social connections that extend beyond the physical education class, fostering a sportsmanlike civic virtue in their daily lives.

## 1 Introduction

In March 2024, a shocking teen murder case in a rural Chinese city (1) brought national attention to the mental health of rural children left behind by migrant workers (thereafter, left-behind children). Criminology theories have long established a link between family support and juvenile delinquency (2, 3). Children require strong bonds within their environment, with the family playing a crucial role in their mental development. Through these familial bonds, children are both protected and influenced by the actions of family members, which can positively or negatively impact their mental health. When supportive conditions are lacking, the likelihood of juvenile crime increases, and such children may develop into serious offenders in adulthood (4). For millions of left-behind children in China, maintaining a close connection with their parents during critical developmental years is often not possible, leading to significant societal issues. Evidence shows that youth bullying in school settings is a widespread issue in rural China (5, 6), with particularly high rates among left-behind children (7). Previous studies commonly report that left-behind children who experience bullying tend to have poor peer relationships and a general dislike for school (5, 8). While it remains unclear whether poor peer relationships lead to bullying or if being bullied results in strained peer interactions, the key point is clear: left-behind children may suffer from social isolation in school, in addition to lacking the traditional parental support essential during their developmental years. This alarming trend calls for immediate societal action to address the mounting concerns regarding the well-being of these vulnerable children. Supportive measures that can reshape children's prosocial behaviors—whether or not they come from traditional family structures—are urgently needed. While traditional psychological counseling is often considered the primary therapy in such situations, mass interventions that promote childhood sportsmanship might offer a novel way to foster citizenship behaviors. However, sportsmanship is not yet widely recognized as an integral component of civilized moral codes. As Keating (9) and we (10) have argued, modern sports represent a manifestation of human nature in contemporary society. The fact that we, as members of a civilized community, can transform violent activities—such as boxing and wrestling—into regulated competitions demonstrates how sportsmanship can internalize societal norms and values through consistent, structured sports education. Therefore, this study will explore the potential of physical education in cultivating a civil mentality in rural children.

Alongside initiatives designed to limit exposure to bullying within campus environments (11), interventions could also benefit from targeting preexisting vulnerabilities, particularly by fostering resilience. Resilience is an individual's ability to adapt positively, recover quickly, and cope flexibly when faced with stress, trauma, or adversity (12). Individuals with higher resilience levels tend to employ proactive coping strategies when facing external challenges and pressures, mitigating adverse effects on mental well-being (13). For example, children and adolescents who had experienced bullying were 3.02 times more likely to develop anxiety symptoms (14). However, the impact of bullying-related anxiety was better managed in victims who demonstrated stronger emotional regulation, interpersonal assistance, and family support—all characteristics of resilience (14). Similar results have also been reported among left-behind children: those exhibiting higher resilience demonstrate a protective effect against developing depression (15). However, Chinese students in rural areas tend to have lower levels of resilience (16), possibly due to socioeconomic inequality and cultural values (17). Furthermore, challenges in family functioning—as is common among left-behind children—could adversely affect resilience (18, 19), resulting in a negative feedback loop characterized by poor adaptation to adversity, diminished social belongings and academic performance, and hindered long-term personal development. This highlights the importance of improving resilience as part of broader efforts to support rural children's development.

Resilience is not solely an inherent trait but also an outcome that can be cultivated and adapted through efforts by families, communities, and schools. Various interventions targeting childhood resilience development have shown promising outcomes (20), which primarily focus on building psychological skills. In contrast, early childhood education theories have long recognized the central role of physical play in optimizing neurocognitive development and enhancing interpersonal adaptation (21). This distinction is critical in the context of mental health development, as psychological counseling is often a passive form of skill acquisition, whereas children learn from personal experience and shape their social beliefs during semi-organized, natural play. While this is not to suggest that one approach should replace the other, the limited empirical evidence on play-focused interventions presents a theoretical opportunity in this field. Hence, regular sports participation emerges as a distinctive solution for enhancing children's resilience, aligning naturally with their developmental patterns. A wealth of literature demonstrates that regular engagement in sports during adolescence serves as a protective factor against mental illnesses (22) and improves resilience (23–25). Furthermore, the type of sports—individual vs. team-based—plays a critical role in shaping mental health outcomes. Team sports, in particular, have been shown to foster resilience-related traits such as stress management and reduced depression symptoms (26). A longitudinal study involving 9,668 individuals exposed to adverse childhood experiences found that participation in team sports during adolescence was associated with better mental health in adulthood, positioning team sports as a scalable tool for resilience building (27). Team sports games, characterized by their team-oriented structure and blend of competitive and recreational elements, integrate the benefits of physical activity with opportunities for social interaction and emotional development. These activities not only resonate with children's innate energy and playfulness but also have the potential to positively influence their social, emotional, and personality development.

A growing body of research has explored the mental health benefits of team sports games. For instance, Wang and colleagues conducted a 10-week intervention with 14 college students diagnosed with moderate clinical anxiety (28). Participants engaged in weekly 2-hour team sports games, leading to significant improvements in mental health, with all participants showing no clinically meaningful anxiety symptoms by the end of the study. Similarly, Liu and colleagues compared the effects of team sports games and individual games on college students with clinical depression (29). The study revealed that integrating team sports games into traditional psychological counseling produced the most substantial treatment effects. More recently, Wang and colleagues examined the impact of team sports games on emotion regulation in kindergarten children (30). Their study implemented 40-min sessions, three times per week, over six weeks, resulting in notable improvements in emotional regulation. Collectively, these preliminary studies highlight the potential of team sports games as a promising intervention for mental health improvement. To our knowledge, however, only two unpublished theses have examined the effectiveness of team sports games on the mental health of Chinese elementary school-aged children. Specifically, within the focus group of rural children, there has been just one study investigating how team sports games could influence their sense of belonging (31). This highlights a significant gap in current research regarding the role of team sports games in building resilience among rural children.

It is worth mentioning that traditional Chinese physical education primarily focuses on sports skills and fitness development. In other words, its strong emphasis on individual development mirrors the approach taken in other academic subjects, where partnership and cooperation for a shared goal receive minimal attention. In contrast, team sports provide a rare opportunity to expand students' social circles and foster common interests. If the aforementioned theoretical background holds, rural children—especially those with less optimal peer relationships—could particularly benefit from such activities by enhancing their interpersonal relationships and, consequently, their resilience. Therefore, the purpose of this study was to evaluate the effects of a semester-long participation in team-building sports games on the resilience of Chinese rural students. The findings are anticipated to provide both theoretical insights and practical recommendations for enhancing the mental health of millions of vulnerable children living in rural areas across China.

## 2 Methods

### 2.1 Exprimental design

This cluster-randomized study was approved by the Ethics Committee of Hunan Normal University and the local Ministry of Education of Nanxian County, China. Assuming a Cohen's *d* of 0.8, a *p*-value of 0.05, and a power of 80% in a two-tailed *t*-test, the minimum number of participants required to demonstrate statistical significance for this pre-post, two-arm design was 26 participants per group (32). Accounting for a potential dropout rate of 10%, a total of 58 participants were needed.

**Figure 1:**
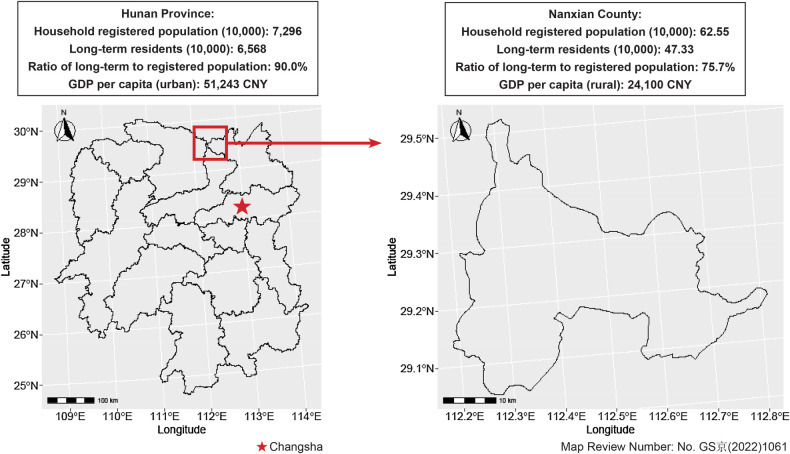
Sampled region. The data are assessed through https://www.hongheiku.com as of December 2024.

Participants were enrolled from an elementary school in Nanxian County, a rural area in Hunan Province. As shown in Figure 1, Nanxian County has a population where 75.7% are long-term residents, with a significant portion of the population, mostly active laborers, working away from their families. For example, Changsha, located less than 90 min away by commercial vehicle, offers more economic opportunities than Nanxian County, which relies primarily on agriculture. Two classes of 10- to 12-year-old fifth-grade elementary school students were enrolled, with each class assigned to either the intervention group or the control group. The intervention group included 43 students (21 boys and 22 girls; mean age, 11.0 years old), while the control group also had 43 students (19 boys and 24 girls; mean age, 11.0 years old). No dropouts or adverse events related to the study occurred, so data from all 86 students were included in the analysis. Participation was voluntary, and written informed consent was obtained from the students' legal guardians prior to their involvement. Of note, 81.4% of parents in the intervention group and 93.0% of parents in the control group reported that one or both parents were employed outside of Nanxian County, either by commuting daily or residing in another city, during the year preceding the study period. According to the State Council's definition, left-behind children are minors under the age of 16 whose parents are either both working away from home or, in cases where only one parent works away, the other parent is unable to provide adequate guardianship. However, the family structure in our sample deviates from the traditional model in which both parents actively contribute to a child's upbringing. Nonetheless, although our sample is not composed entirely of left-behind children as defined by the State Council, it is drawn exclusively from rural areas, which aligns with the study's focus on the resilience of children from the lower end of social spectrum.

### 2.2 Intervention

The study was conducted during the Spring 2024 semester. From March 1 to June 1, participants engaged in a 12-week intervention as part of their regular physical education classes, held three times per week. For the intervention group, the 15-min warm-up period was replaced with team-building games, as shown in Table 1. In addition to ensuring minimal equipment requirements, these games were selected based on two fundamental principles that contribute to the development of childhood resilience. First, games that involve physical touch were prioritized. According to John Bowlby's attachment theory (33), positive physical contact is essential for forming healthy attachments. A wealth of evidence supports not only the role of touch in social-emotional development and affect regulation (34) but also its contribution to a range of positive physiological responses (35). Rural children, who often face academic pressures and may lack sufficient parental care, could particularly benefit from engaging in physical contact with peers during gameplay. This interaction helps shape their emotional exchanges in a relaxed environment. Consequently, games such as “apply a medicated patch” and “fishing” were included because they involve age-appropriate physical contact. Second, these games are designed to foster team awareness and cooperation. Each game requires a degree of collaboration that aims to help students build new social connections beyond the playing field. In contrast, the control group's warm-up period consisted of standard activities such as jogging and stretching. After the warm-up, both groups participated in identical sports activities based on China's Compulsory Education Physical Education and Health Curriculum Standards. To minimize potential environmental and instructional biases, an independent physical education teacher, trained to instruct the sports games, conducted all classes for both groups using the same fields, venues, and main class content.

**Table 1:** Schedule of team-building sports games over the intervention period.

Week	Game	Week	Game	Week	Game
1	Counting and forming groups	5	Tag	9	Ball toss and count
2	House, rabbit, earthquake	6	Obstacle relay race	10	Dragon biting its tail
3	Apply a medicated patch	7	Three-legged race	11	Defend the earth
4	Fishing	8	Line stepping chase	12	Tug of war

The full instructions for each game, including the original Chinese and English translations, can be accessed on Figshare (https://doi.org/10.6084/m9.figshare.28092509.v1). The manual (CC BY 4.0) for these sports games can be freely distributed and modified for teaching purposes.

### 2.3 Instrument

Resilience was assessed using the Resilience Scale for Chinese Adolescents (36), as shown in Table 2. The scale is widely recognized in adolescent psychology research in China and has been used in previous studies to assess resilience among rural students (37). For example, the item “When facing difficulties, I focus all my energy” conveys determination and a strong commitment to addressing challenges. It reflects a focused mindset and the ability to channel resources and efforts toward overcoming obstacles. This 27-item scale evaluates five factors of resilience: goal planning, affect control, positive thinking, family support, and help-seeking. Each item is rated on a 5-point Likert scale, with higher scores for each factor indicating greater resilience. By combining the scores of the first three factors—goal planning, affect control, and positive thinking—we derive a higher-order factor called “individual power”, which reflects an adolescent's inherent resilience capacity. Similarly, aggregating the scores of the family support and help-seeking factors produces a higher-order factor called “supportive power”, indicating the additional resilience gained through support from others. In this study, the scale demonstrated a Cronbach's α of 0.827 and a KMO value of 0.746, confirming its reliability and validity.

**Table 2:** English translation of the resilience scale for Chinese adolescents.

Goal planning
My life has clear goals.
After facing setbacks, I typically emerge more mature and experienced.
When faced with difficulties, I usually create a plan and develop solutions.
When facing difficulties, I focus all my energy.
I set goals for myself to drive progress and stay motivated.
Affect control
Failure often leaves me feeling discouraged.^a^
I find it challenging to manage my negative emotions.^a^
Failure and setbacks often lead me to doubt my abilities.^a^
I often take a long time to move on from unpleasant experiences.^a^
I can quickly adjust my emotions in a short period of time.
My emotions tend to fluctuate significantly, and I am prone to extreme highs and lows.^a^
Positive thinking
I believe that the process of doing something contributes more to personal growth than the outcome.
I believe that adversity can have an inspiring effect on people.
Adversity can sometimes serve as a catalyst for growth.
I believe that every situation has a positive aspect.
Family support
My parents value my opinions.
My parents often interfere with my thoughts.^a^
At home, it feels like no one ever listens to what I say.^a^
My parents lack confidence in me and fail to provide emotional support.^a^
My parents never criticize me harshly.
My parents always encourage me to put forth my best effort.
Help-seeking
When I encounter unpleasant situations, I often struggle to find the right person to talk to.^a^
I have a peer with whom I can share my difficulties.
When I face difficulties and need help, I often don't know who to turn to.^a^
I tend to keep things bottled up inside rather than talking to others.^a^
When I face difficulties, I actively seek out others to talk to.
When I'm feeling down, I prefer not to talk to others about it.^a^

^a^ Denotes reverse-scored items.

The assessment was conducted one day before the first intervention session and one day after the last session, using paper-and-pencil methods in a classroom setting. One researcher (B.L.) was present during both sessions to explain the survey to the participants and to answer any questions before the survey was completed. Of note, before the study began, each participant was assigned a unique ID, known only to a single researcher (Y.X.), who was not involved in the statistical analysis. Participants completed the scale anonymously to minimize social desirability bias. The completed surveys were analyzed by another researcher (B.L.), who had access only to the unique IDs and no other identifying information.

### 2.4 Statistics

The complete data supporting the conclusions of this study are available on Figshare (https://doi.org/10.6084/m9.figshare.28092509.v1). All statistical analyses were conducted using R version 4.4.2 (Pile of Leaves). This study employed a cluster randomized controlled design with two assessments conducted before and after the intervention. Due to the nature of the questionnaire data, various transformation methods were attempted to meet statistical assumptions, including log transformation, Yeo-Johnson power transformation, and the inverse hyperbolic sine transformation. However, none of these methods yielded satisfactory results. Consequently, the Mann–Whitney *U*-test was used to compare group and/or time differences for nonparametric data. For Gaussian data, mixed analysis of variance was employed to assess group and/or time differences. When a significant interaction effect was detected, Welch's *t*-test was applied for simple main effects, focusing on the between-group main effect. A two-sided *p*-value of 0.05 was set as the threshold for statistical significance.

To enhance the dissemination of research findings, *p*-values were converted into probabilistic-based common language effect size (CLES) (38) or its nonparametric equivalent, Ruscio's *A* statistic (39). In brief, practitioners (e.g., physical education teachers) may not always be familiar with traditional statistical metrics like Cohen's *d* or *p*-values. For instance, if a research paper reports a small effect size with a Cohen's *d* of 0.2, someone without a statistical background might assume that the intervention might be useful, albeit small. However, when translated into common language using probabilistic terms, a Cohen's *d* of 0.2 means there is only a 55.6% chance that a person receiving the treatment will outperform someone in the control group. In other words, the outcome is almost as likely as a 50–50 chance, which hardly supports the intervention's effectiveness. Therefore, expressing effect sizes in common language not only preserves the traditional notion of statistical significance but also conveys a clearer and more intuitive understanding of the intervention's effectiveness. These conversion can be performed using Equation (1):

(1) $$\begin{array}{rl} & \mathrm{CLES}\;=\;\Phi \left(\frac{d}{\sqrt{2}}\right)\\ & A\;=\;\frac{n_{1}\times n_{2}-U}{n_{1}\times n_{2}}, \end{array}$$

where, Ф is the normal cumulative distribution function, *d* represents the Cohen's *d*, n_1_ and n_2_ denote the sample sizes for each group, and *U* is the Mann–Whitney *U* statistic.

## 3 Results

**Figure 2:**
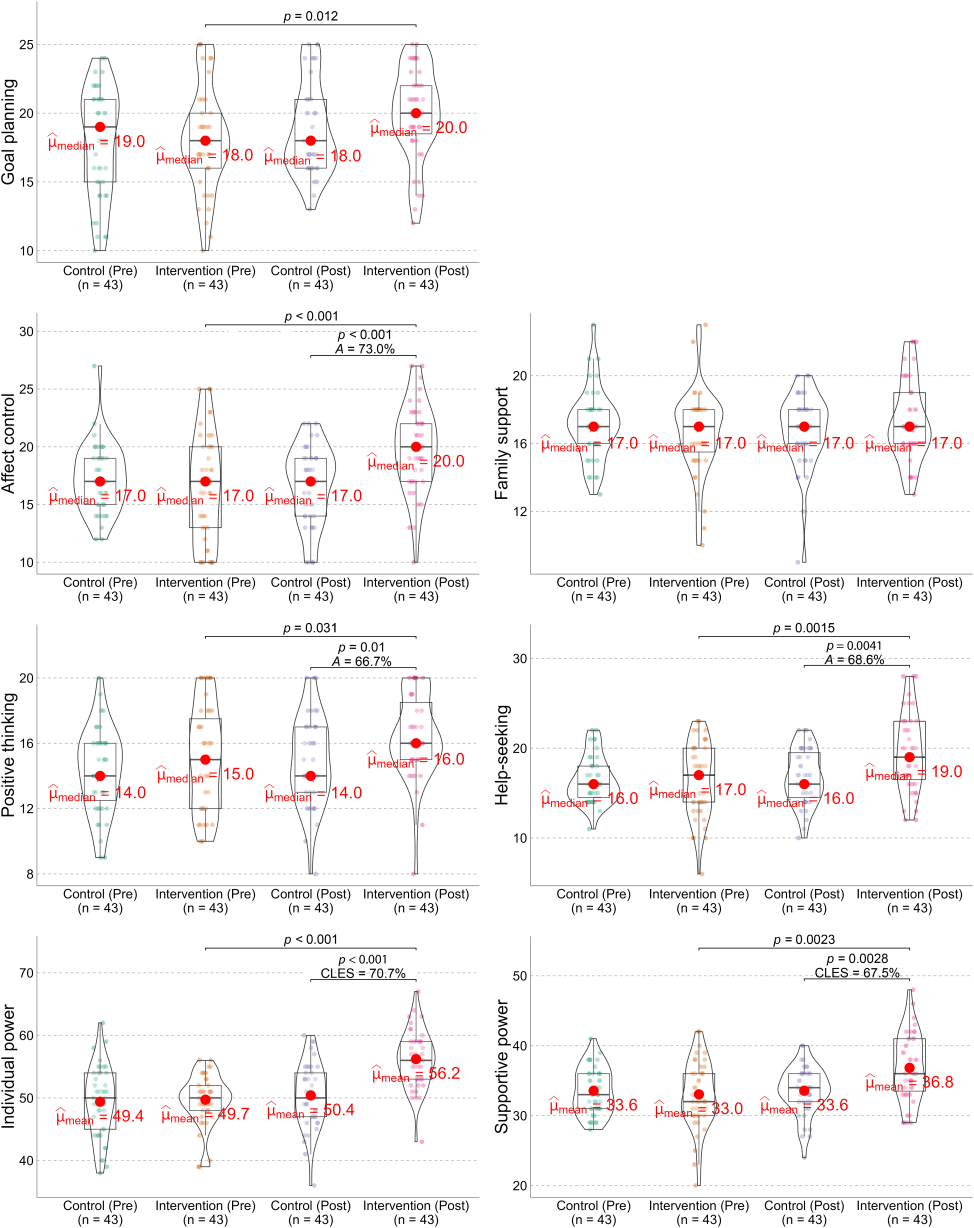
Effect of 12-week team-building sports games on the resilience of rural elementary school children. Data are visualized using box-violin plots, which display the density, spread, and outliers of the data. The measure of central tendency (û) is chosen according to the data's distribution. The between-group differences at the study's conclusion are quantified using the probabilistic statistics. CLES, common language effect size. *A*, Ruscio's *A* statistic.

Figure 2 illustrates the change in resilience over a 12-week intervention period. With respect to the goal planning factor, although the intervention group showed a significant within-group improvement (*p* = 0.012), no significant difference was observed between the intervention and control groups at the conclusion of the study (*p* > 0.05). Therefore, it cannot be concluded that the 12-week team-building sports games improved goal planning in this sample. However, the intervention group showed significant improvements in affect control and positive thinking compared to the control group. According to the probabilistic measure, 10- to 12-year-old children who participated in the 12-week team-building sports games had a 73.0% chance of outperforming their peers in the control group in affect control. Similarly, children in the intervention group had a 66.7% probability of outperforming those in the control group in positive thinking. Consequently, the individual power of resilience significantly improved after the 12-week team-building sports games, with an overall chance of 70.7%.

No significant difference was observed between the groups for the family support factor. However, the help-seeking factor showed significant improvement. Children who participated in the intervention had a 68.6% chance of outperforming those in the control group in help-seeking. When combined, the supportive power of resilience in the intervention group was significantly improved compared to the control group, with an overall chance of 67.5%.

## 4 Discussion

According to Article 31 of the United Nations Convention on the Rights of the Child (40), play is recognized as a fundamental need and right for children. Hence, access to age-appropriate sports games is an essential component of children's rights in school, supporting their normal development. In contrast to similarly aged children in resource-rich, developed economies, many enjoyable activities—such as games and extracurricular interests—are unfortunately squeezed out of the daily lives of Chinese children due to the heavy burden of academic work (41). Without effective mechanisms to alleviate this pressure, Chinese children face an increasing prevalence of depression (42), which can exacerbate mental health challenges and potentially lead to criminal behaviors later in life. If major reforms to the examine-focused education system—widely regarded as the only credible route to social mobility—do not materialize, then physical education classes become the sole opportunity for school educators to offer students enjoyable social interactions with peers during the regular school day. With these concerns in mind, this study empirically investigated whether replacing traditional warm-ups in physical education classes with team-building sports games could positively influence children's resilience. After approximately one semester of intervention, children in the intervention group exhibited improvements in affect control, positive thinking, and help-seeking behaviors—findings that align with previous research on the benefits of physical activity for enhancing rural children's resilience (37).

The mechanism through which team-building sports games influence the resilience of rural children may involve a combination of factors. First, strong evidence supports the role of team sports in promoting affect control and positive thinking among athletes (43, 44). In general, joining a sports team shifts an individual's focus toward two main objectives: personal interests and team interests, both of which converge toward a common outcome. As a result, athletes learn to regulate both their own emotions and those of others (e.g., as a team captain) for the benefit of the team (45). Moreover, improved emotion management—including enhanced self-regulation and better interpersonal experiences—has been linked to superior performance (46), creating a positive feedback loop that further reinforces an individual's affect control. During team-building sports games, children experience a variety of emotions—from the excitement of victory to the frustration of failure and the tension of facing strong opponents. Successfully overcoming challenges through sound decision-making builds both self-efficacy and team awareness. Even when faced with failure, students learn to confront difficulties and persist through setbacks. The present results provide new evidence supporting the theory that even regular, brief participation in team-building sports games can serve as a novel catalyst for shaping the emotional development of young minds.

Second, improvements in help-seeking behaviors among children may be attributed to the inherent competitive nature of sports, the intentional design of the games, or a combination of both factors. The competitive aspect of sports stimulates a desire to excel, while effective cooperation and communication are essential for team success (47). In these settings, team members must work together to develop strategies for completing game tasks, a process that enhances social skills, fosters a sense of belonging, and encourages mutual support. Additionally, this study deliberately selected contact-based sports games to serve as a catalyst for touch-stimulated social experiences (48). In our view, physical contact is an often overlooked yet crucial component of early childhood education, and its inclusion represents a novel contribution of the present study. For instance, in adult social interactions, handshakes are pivotal in shaping self-presentation, assessing others, and building interpersonal relationships. Similarly, touching pets can evoke empathy and promote social bonding across species (49). The physical contact inherent in these sports games not only facilitates cooperative interactions but may also help reduce the invisible psychological distance experienced by children who tend to self-isolate. As a result, the sports games foster new peer attachments that encourage help-seeking when facing life's difficulties, benefiting all involved. This novel perspective warrants further investigation using objective measures, such as those provided by neuroscience.

While the primary objective of physical education is to develop students' physical capacity during their growth, we argue that its ultimate goal should be to foster lifelong exercise habits that enhance both physical and mental health, thereby contributing to the development of all-rounded individuals and a competitive nation (10). The findings from this study provide evidence that team-focused physical education can serve as a strategic platform for mental health promotion—especially in settings where children face intense academic pressures, limited opportunities for social engagement outside the classroom, and inadequate parental care. Chinese policymakers should recognize that play is a fundamental right of school-aged children. Our results show that even a semester-long 15-min game play can foster positive resilience development. However, the current national curriculum only allocates three 45-min physical education classes per week, which may be insufficient to fully mitigate the negative impacts of academic stress. By incorporating daily, purposefully designed, and engaging physical education classes, schools have a unique opportunity to support the holistic development of their students. Consequently, policymakers and educators are encouraged to explore and implement sports interventions similar to those in this study as part of a strategy to promote physically, mentally, and academically balanced development, particularly among rural children.

We acknowledge several limitations here. Given the diverse socioeconomic backgrounds in China, research involving diverse populations is needed to replicate these findings. Additionally, because the experimental period lasted only one semester, it remains unclear whether the observed improvements will persist over time. To our knowledge, resilience is generally viewed as a dynamic process rather than a permanent, unchanging trait—even though resilient children tend to exhibit better mental health into adulthood (50). In other words, it is uncertain whether the improvements in the resilience of rural children observed in this study reflect acute psychological responses or longer-term adaptation. Nonetheless, even if a child has developed a high level of resilience, maintaining it may require ongoing effort and supportive conditions. Therefore, longitudinal studies are warranted to better understand the long-term effects of current interventions on resilience, particularly whether these improvements persist as a stable trait over time.

Finally, the lack of improvement in the goal planning and family support domains opens new avenues for research. These results were expected given that the games were designed with clear, straightforward objectives that required minimal planning and did not incorporate family-related factors. Future research could specifically target these two aspects to offer children more engaging experiences that enhance goal planning skills and integrate family elements. When designing the games, we ensured that they were universally accessible in economically disadvantaged areas by relying on commonly available equipment. For schools in more affluent cities, however, there is an opportunity to incorporate advanced equipment and design more complex, goal-oriented sports games. For example, integrating puzzles and obstacles that require children to devise efficient routes to reach a finish line could effectively promote goal planning. Regarding the family element, emerging telehealth platforms present promising possibilities. Such platforms could facilitate virtual group exercises that allow children and their parents to engage in physical activities “together”. This approach could achieve dual objectives: encouraging more physical activity for both parents and children, and providing a virtual space that helps mitigate the lack of direct parental care for left-behind children.

In conclusion, a 12-week team-building sports games intervention enhanced the resilience of rural elementary school students aged 10–12. The students demonstrated improved affect control, more positive thinking, and a greater willingness to seek help. These traits are likely to yield long-term benefits throughout their growth and into adulthood. Our study provides preliminary evidence that revising physical education curricula in China to include engaging, hands-on sports games could build resilience and promote the holistic development of children.

## Data Availability

The datasets presented in this study can be found in online repositories. The names of the repository/repositories and accession number(s) can be found in the article/Supplementary Material.
